# The Impact of Mitt and Traditional Restraint Use on Length of Stay in Hospital Dementia Care

**DOI:** 10.1093/geroni/igaf122.2195

**Published:** 2025-12-31

**Authors:** Clarissa Shaw, Gregory Isaac, Lisa Weimar, Caitlin Ward

**Affiliations:** University of Iowa College of Nursing, Iowa City, Iowa, United States; University of Minnesota School of Public Health, Minneapolis, Minnesota, United States; University of Iowa, Iowa City, Iowa, United States; University of Minnesota School of Public Health, Minneapolis, Minnesota, United States

## Abstract

The regulation of physical restraints in dementia care varies between hospitals and nursing homes, creating a gap in how devices like mitts are classified. Mitts are padded gloves that prevent finger and thumb movement. Traditional physical restraints are wrist, ankle, or vest devices that secure a patient to their bed or chair. While nursing homes classify mitts as restraints, hospital practices vary widely, with some designating them as “restraint alternatives” that do not require a provider order or frequent monitoring. This regulatory discrepancy creates discharge barriers for patients in mitts, as many nursing homes cannot accept patients that are actively restrained, potentially prolonging hospitalizations. This study examined whether mitt use impacts length of stay similarly to traditional restraints. A retrospective analysis of electronic medical record data from a single university hospital that treats mitts as restraint alternatives was conducted. The data included 1,503 patients with dementia admitted to medical-surgical units across 2,141 hospital stays with an average length of stay of 8.3 days. Mitts were utilized in 15.9% of hospital stays and traditional physical restraints were used in 12.5% of stays. Linear mixed modeling revealed that mitt use was associated with a 4.4-day increase in length of stay (SE = 0.29, p<.001) compared to patients without mitts. Similarly, traditional restraint use was associated with a 3.9-day increase (SE = 0.33, p<.001) compared to patients without traditional restraints. Despite having comparable impacts on length of stay as traditional restraints, mitts remain under-recognized in hospital settings while being regulated as restraints in nursing homes.

